# The Secret Life of Tethers: The Role of Tethering Factors in SNARE Complex Regulation

**DOI:** 10.3389/fcell.2016.00042

**Published:** 2016-05-09

**Authors:** Michelle L. Dubuke, Mary Munson

**Affiliations:** Department of Biochemistry and Molecular Pharmacology, University of Massachusetts Medical SchoolWorcester, MA USA

**Keywords:** intracellular trafficking, multisubunit tethering complexes, SNARE complexes, Sec1/Munc18

## Abstract

Trafficking in eukaryotic cells is a tightly regulated process to ensure correct cargo delivery to the proper destination organelle or plasma membrane. In this review, we focus on how the vesicle fusion machinery, the SNARE complex, is regulated by the interplay of the multisubunit tethering complexes (MTC) with the SNAREs and Sec1/Munc18 (SM) proteins. Although these factors are used in different stages of membrane trafficking, e.g., Golgi to plasma membrane transport vs. vacuolar fusion, and in a variety of diverse eukaryotic cell types, many commonalities between their functions are being revealed. We explore the various protein-protein interactions and findings from functional reconstitution studies in order to highlight both their common features and the differences in their modes of regulation. These studies serve as a starting point for mechanistic explorations in other systems.

## SNARE complexes

Transfer of protein and lipid cargo between distinct cellular compartments in eukaryotes is achieved through the use of membrane-bound vesicles. These vesicles bud from a donor compartment, are trafficked to and then fuse with their target membrane. Several conserved protein families have evolved to control these various processes, with specificity for particular trafficking pathways. The core of the membrane fusion apparatus is the SNARE complex (Jahn and Scheller, 2006); to facilitate fusion, each set of membranes has a distinct subset of SNARE proteins—t-SNARE proteins on the target membrane, and v-SNARE proteins on the vesicle membrane. Formation of this extremely stable SNARE complex is sufficient to overcome the energy barrier to membrane fusion, and is sufficient for fusion in simplified *in vitro* systems (Weber et al., 1998; Brünger et al., 2015). Additionally, mutations in individual SNARE proteins, changes in SNARE protein levels, and changes in SNARE complex assembly all result in various cellular and organismal defects from yeast through humans, indicating the necessity of these proteins in all stages of trafficking (Johnson et al., 2008; Garcia-Reitböck et al., 2010; Kama et al., 2011).

The regulated fusion of vesicles at proper target membranes requires specific recognition factors on both membranes. Several lines of evidence indicate that SNAREs alone are not sufficient for specificity. First, SNAREs can form stable, fusion-competent non-cognate complexes *in vitro* (Fasshauer et al., 1999; Yang et al., 1999; McNew et al., 2000; Bethani et al., 2007; Furukawa and Mima, 2014). Secondly, at least for exocytosis, protein localization is not sufficient to promote specific SNARE pairing, as many SNAREs are not specifically restricted to sites of active membrane fusion (Brennwald et al., 1994; Jahn and Südhof, 1999). Furthermore, SNAREs need to traffic through the secretory pathway to reach their final destination, and encounter many cognate and non-cognate SNAREs along the way. When this process is not properly controlled, e.g., failing to inhibit SNARE complexes prior to trafficking, the proteins can form non-fusogenic complexes early in the secretory pathway (Medine et al., 2007). Therefore, to prevent premature or inappropriately localized fusion, SNARE proteins must be tightly controlled. Many syntaxin t-SNARE family members contain an autoinhibitory domain that prevents premature complex assembly (Weimbs et al., 1997; Nicholson et al., 1998; Munson et al., 2000; Dietrich et al., 2003). This and other mechanisms of inhibition and subsequent activation are likely provided by the protein families that are properly localized at sites of fusion, such as the Sec1/Munc18 (SM) proteins and the tethering factors.

## SM proteins

The SM proteins are a family of SNARE regulators with diverse and (at times) seemingly contradictory functions (Carr and Rizo, 2010; Archbold et al., 2014). Many different protein-protein and genetic interactions were identified for each of the four SM family members (Sec1/Munc-18, Vps45, Sly1, and Vps33). SM proteins interact with SNAREs using a number of different binding modes, often utilizing more than one binding mode simultaneously. The functional consequences of these interactions include regulating protein levels of partner SNAREs, promoting SNARE assembly and downstream membrane fusion, chaperoning/stabilizing the autoinhibited conformation of syntaxin prior to SNARE complex assembly, and working with the Sec17/Sec18 (NSF/SNAP) SNARE disassembly machinery to protect cognate SNARE complexes from disassembly prior to fusion (Carr et al., 1999; Sato et al., 2000; Bryant and James, 2003; Kennedy et al., 2004; Deák et al., 2009; Hashizume et al., 2009; Struthers et al., 2009; Lobingier and Merz, 2012; Lobingier et al., 2014). Most SM proteins also interact with tethering complexes, either transiently or as a stoichiometric component of the intact complex (discussed below). The most compelling mechanistic details of SM protein function were published recently, for the vacuolar fusion SM protein Vps33 (a component of the HOPS tethering complex): a pair of crystal structures suggested that if Nyv1 (v-SNARE) and Vam3 (t-SNARE) interact simultaneously with Vps33, these SNARE proteins would be correctly aligned to initiate zippering of the SNARE complex (Baker et al., 2015).

## Tethering complexes

**Figure 1:**
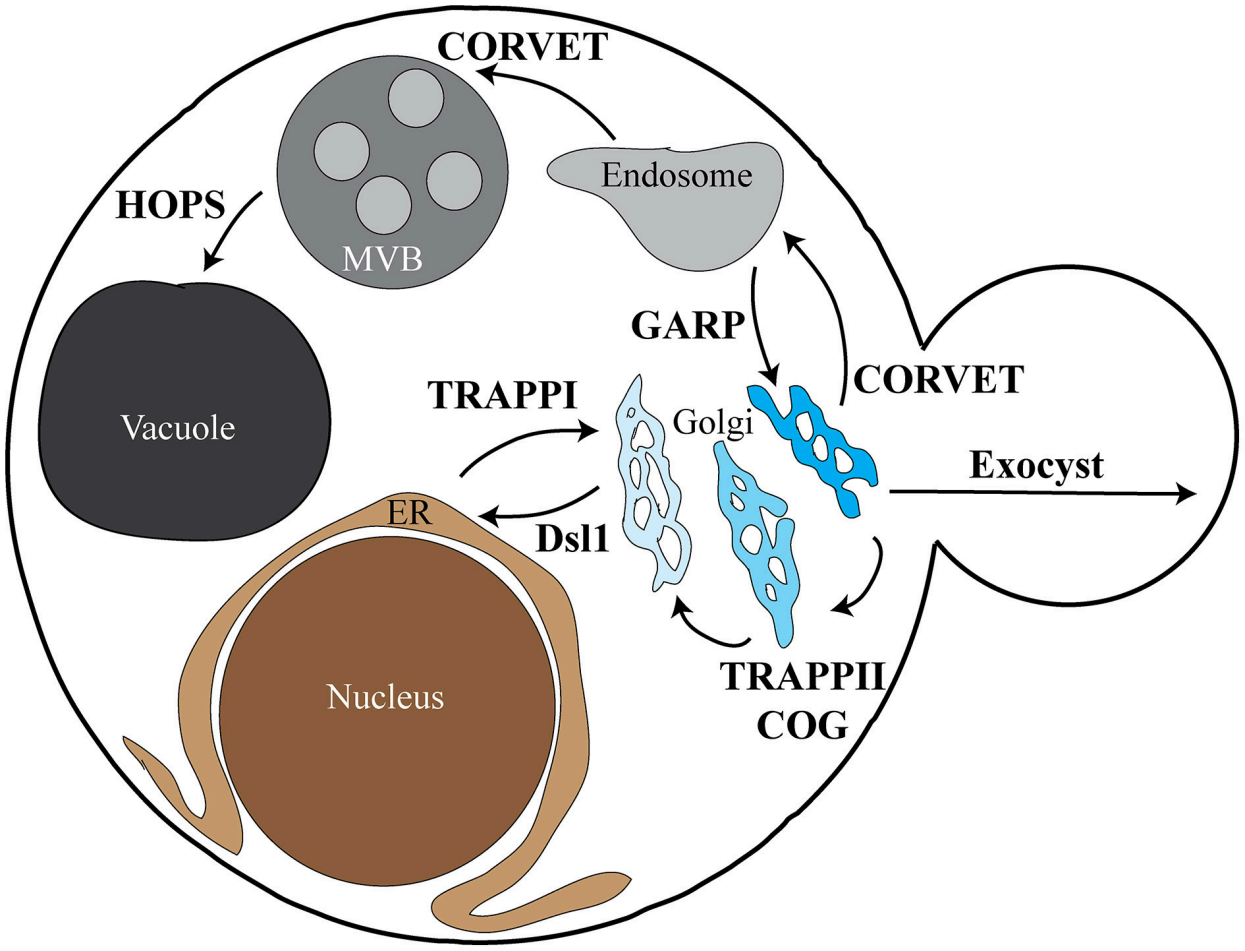
**Overview depicting MTC localization in yeast**. The various subcellular locations and trafficking pathways in yeast are depicted, along with the tethering complexes associated with each pathway. The TRAPPI complex is involved in ER to cis-Golgi traffic, with the Dsl1 complex required for retrograde Golgi to ER traffic. The TRAPPII and COG complexes are involved in retrograde Golgi traffic between the various Golgi compartments. CORVET functions in both trans-Golgi to early endosome trafficking and early endosome to MVB/late endosome trafficking. The HOPS complex is required for MVB/late endosome to vacuole/lysosome vesicle fusion. Early endosome to Golgi recycling requires the GARP complex, while Golgi to plasma membrane trafficking requires the exocyst complex. Each of the pathways depicted have associated SNARE proteins; the tether-SNARE interactions discussed in this review are outlined in Table 1. The role of TRAPPIII in autophagosome formation is not shown. ER, endoplasmic reticulum; MVB, multivesicular body/late endosome.

SNARE complex assembly and fusion are also regulated by the multisubunit tethering complexes (MTCs; Bröcker et al., 2010; Yu and Hughson, 2010; Hong and Lev, 2014). The MTCs are compartment-specific complexes proposed to promote the initial interaction between a vesicle and its target membrane via interactions with lipids and proteins on both membranes. These interactions are critical to promote specific vesicle fusion at the proper target destinations. Most steps of membrane trafficking are associated with an MTC (Figure 1), and while these complexes differ in subunit composition and number they each interact with the same families of proteins—Rab GTPases, SM proteins, coat proteins, and SNAREs (Table 1). Many MTCs also interact with the specific Guanine nucleotide Exchange Factor (GEF) for their GTPases. These complexes function upstream of SNARE assembly and vesicle fusion, resulting in vesicle accumulation at their sites of fusion upon disruption of MTCs. Although we focus on the MTCs in this review, the long coiled-coil tethers have also been implicated in SNARE complex regulation (Cheung and Pfeffer, 2016).

**Table 1:** **Summary of MTC-SNARE interactions**.

**Function**	**Tethering complex**	**SNARE Interaction**	**References**
Localization of MTC/SNAREs	HOPS	Vam7	Wang et al., 2003
			Zick and Wickner, 2013
	COG	Unknown partners	Oka et al., 2004
			Willett et al., 2013a
	Exocyst	Snc2	Shen et al., 2013
Protect SNARE complex from disassembly	HOPS	Pre-fusion SNARE complexes	Collins et al., 2005
			Collins and Wickner, 2007
			Xu et al., 2010
	HOPS	Properly assembled SNARE complexes	Starai et al., 2008
	COG^*^	Assembled SNARE complexes	Shestakova et al., 2007
	Exocyst^*^	Assembled SNARE complexes	Dubuke et al., 2015
	GARP^*^	Assembled SNARE complexes	Siniossoglou and Pelham, 2002
Promote SNARE assembly/Stabilize SNARE proteins	COG	Unknown partners	Fotso et al., 2005
			Oka et al., 2004
			Shestakova et al., 2007
	GARP	Unknown partners	Siniossoglou and Pelham, 2002
	Dsl1	Unknown partners	Meiringer et al., 2011
Unknown Function(s)	CORVET	Pep12 (t-SNARE)	Subramanian et al., 2004
		Assembled SNARE complexes	Balderhaar et al., 2013
	Dsl1	Sec22/Sec20	Kraynack et al., 2005
	GARP	Tlg1	Conibear et al., 2003
			Siniossoglou and Pelham, 2001
		SNARE domains of Stx6, Stx16 and Vamp4	Pérez-Victoria and Bonifacino, 2009

The functional implications of the various tether-SNARE interactions in yeast and mammalian cells are summarized. The

* *indicates an extrapolation of the functional consequence of the interaction for that tethering complex based on similar interactions in other stages of trafficking. For COG, GARP, and Dsl1, functional effects of SNARE interactions were observed, but the specific SNAREs involved have not been identified. Conversely, other specific interactions with SNAREs were identified for CORVET, Dsl1, and GARP, but the functional consequences of those interactions are currently unknown*.

Despite differences in overall complex architecture, a subset of tethering complexes was defined based on the structures of the individual subunits—the Complexes Associated with Tethering Containing Helical Rods (CATCHR) family (Yu and Hughson, 2010). As the name suggests, the proteins in these complexes are composed of long rods of stacked helical bundles. The similar bundle topology of each subunit suggests divergent evolution from an ancient ancestor (Sivaram et al., 2006; Croteau et al., 2009; Yu and Hughson, 2010; Klinger et al., 2013). The remaining “non-CATCHR” complexes have a more diverse subunit composition; however, they still share many of the same protein family interactions, suggesting a common mechanism of action across all of the complexes.

## Dsl1 complex

The CATCHR family Dsl1 complex is localized at the ER and is required for specific recognition of COPI coated retrograde Golgi-derived vesicles prior to fusion with the ER (Andag et al., 2001; Reilly et al., 2001). This complex is the smallest of all known tethering complexes with only three core subunits—Dsl1, Tip20, and Dsl3/Sec39 in yeast (Ren et al., 2009; Spang, 2012) and NAG, RINT1, and ZW10 (NRZ) in mammals (Tagaya et al., 2014). Although the Dsl1 complex is small, it participates in many of the same interactions as the other MTCs. Interestingly the complex does not appear to interact with GTPases on either membrane. Instead, the Dsl1 subunit interacts with the COPI coat of the incoming vesicle, while the Dsl3 and Tip20 subunits interact with SNARE proteins on the target membrane (Zink et al., 2009). While the switching action of a GTPase may not be used to modulate the interaction with the vesicle, the un-coating of the vesicle may provide a similar mechanism for proceeding to SNARE complex assembly (Zink et al., 2009). The complex also interacts with the SM protein Sly1, which functions at the Golgi, although the role of this interaction is unknown (Kraynack et al., 2005).

## GARP complex

The Golgi Associated Retrograde Protein (GARP) complex is a CATCHR family MTC (Bonifacino and Hierro, 2011) required for protein sorting at the late Golgi (Conibear and Stevens, 2000). This complex functions in mammalian cells in two forms—GARP containing Vps54, and EARP containing syndetin. EARP is involved in the recycling endocytic pathway (Schindler et al., 2015).

GARP is another small tethering complex, containing only 4 core subunits. As observed for most MTCs, GARP interacts with a Golgi-localized GTPase, and GARP subunits interact with vesicles through interactions with an as yet unidentified protein (Siniossoglou and Pelham, 2001; Siniossoglou, 2005). Similarly, no physical interaction has been identified between GARP subunits and an SM protein, although a synthetic genetic interaction was identified between the Vps53 subunit and Sly1 (VanRheenen et al., 2001). GARP also has not been shown to physically interact with any GEFs, but several studies identified synthetic genetic interactions between components of GARP and the Ric1/Rgp1 dimeric GEF (Tong, 2004; Costanzo et al., 2010; Hoppins et al., 2011).

## COG complex

The Conserved Oligomeric Golgi (COG) complex is necessary for retrograde transport between Golgi compartments, and is one of the best studied CATCHR complexes. It is composed of 8 subunits that form two separate “lobes” connected through the COG1 and COG8 subunits (Willett et al., 2013b). Additionally, depletion of various COG subunits results in an accumulation of vesicles at the Golgi (Wuestehube et al., 1996; Zolov and Lupashin, 2005). Results from cell-free systems suggested that COG can tether vesicles prior to fusion, supporting a direct role in tethering (Cottam et al., 2013).

To provide its tethering function, both the yeast and mammalian COG complexes interact with small GTPases on both the vesicle and target membranes (Suvorova et al., 2002; Fukuda et al., 2008; Yu et al., 2008; Miller et al., 2013). The COG4 subunit also interacts with the SM protein Sly1 (Laufman et al., 2009), and several COG subunits genetically interact with the GEF Ric1 (Tong, 2004; Schuldiner et al., 2005; Costanzo et al., 2011; Hoppins et al., 2011).

## Exocyst complex

The exocyst complex is the CATCHR-family MTC proposed to recognize and tether secretory vesicles to the plasma membrane (Heider and Munson, 2012; Wu and Guo, 2015). Similar to COG, the exocyst is composed of 8 subunits that form two separate “modules” connected by multiple protein-protein interactions (Heider et al., 2016). Temperature-sensitive yeast exocyst mutants result in vesicle accumulation in the bud, supporting a tethering role upstream of vesicle fusion with the plasma membrane (Novick et al., 1980; Grote et al., 2000).

The exocyst shares many similar interactions with other MTCs, including interactions with lipids and Rab/Rho family GTPases on the vesicle and plasma membranes, although tethering has not yet been directly demonstrated (Adamo et al., 1999; Guo et al., 1999; Zhang et al., 2001, 2008; He et al., 2007; Baek et al., 2010; Wu et al., 2010; Yamashita et al., 2010; Brunet and Sacher, 2014). The exocyst also interacts with the SM protein Sec1 through its Sec6 subunit (Wiederkehr et al., 2004; Morgera et al., 2012). Furthermore, the exocyst interacts with Sec2, the GEF for the vesicle-specific GTPase Sec4, through its Sec15 subunit (Medkova et al., 2006).

## HOPS and CORVET complexes

The non-CATCHR Homotypic Fusion and Vacuolar Protein Sorting (HOPS) Complex and Class C Core Vacuolar/Endosomal Tethering (CORVET) complexes are required for early endosomal homotypic fusion, early to late endosomal fusion, and vacuolar/lysosomal fusion. These subcellular compartments and tethering complexes can be purified; therefore, these trafficking steps can be reconstituted *in vitro* and are well-characterized (Conradt et al., 1992; Haas, 1995; Stroupe et al., 2006, 2009; Hickey and Wickner, 2010; Ostrowski et al., 2010; Plemel et al., 2011; Balderhaar et al., 2013). They are also the only tethering factors shown to be *bona fide* tethers (Brunet and Sacher, 2014).

The architectures of HOPS and CORVET are composed of a shared core of four subunits; the two additional subunits promote binding to specific Rab GTPases (Price et al., 2000; Seals et al., 2000; Peplowska et al., 2007). Unlike other tethering factors, HOPS/CORVET incorporate the SM protein (Vps33) into the complex rather than recruiting it as needed (Seals et al., 2000). It is through this subunit that many of the SNARE interactions occur. One of the HOPS-specific subunits appears to function as a GEF for Ypt7 in yeast, and the HOPS complex interacts with the Ccz1/Mon1 GEF (Wurmser et al., 2000; Nordmann et al., 2010); however, in mammalian systems no evidence of GEF activity has been detected (Peralta et al., 2010). These interaction similarities suggest that HOPS and CORVET, although distinct in structure from other MTCs, share conserved functions (Bröcker et al., 2012).

## TRAPP complexes

The Transport Protein Particle (TRAPP) complexes, of which there are three identified in yeast, are putative tethering factors that function in ER to Golgi transport (TRAPPI), intra-Golgi trafficking (TRAPPII), and autophagosome formation (TRAPPIII; Barrowman et al., 2010; Kim et al., 2016). The TRAPP complexes share the least sequence and structural similarity with the other tethering complexes (Cai et al., 2008). They interact with coat proteins, and specific subunits appear to differentiate ER-derived COPII coated vesicles from the Golgi-derived COPI coated vesicles (Sacher et al., 2001). Additionally, TRAPPI and TRAPPII can function as a GEF for Ypt1, a small Rab GTPase found on ER-derived vesicles and required for fusion with the Golgi (Wang et al., 2000). However, no interactions have been identified between TRAPP and either an SM protein or SNARE proteins/complexes, suggesting that it may function differently than other MTCs.

## MTC-SNARE complex interactions

Interactions between tethering factors and SNARE complexes have been observed for almost all trafficking steps (Table 1), and generally serve to promote formation of proper and stable SNARE complexes (Hong and Lev, 2014; Kuhlee et al., 2015). However, detailed mechanistic studies are needed to understand how these interactions regulate SNARE complex assembly, and the functional roles these interactions play in intracellular trafficking. Furthermore, the relationships between tethers and SM proteins in SNARE regulation remain to be elucidated.

One role for the MTC:SNARE interaction is in promoting MTC localization, SNARE protein localization, or both. For example, HOPS recruitment by the SNARE Vam7 maintains proper HOPS localization at sites of vacuole fusion, and loss of Vam7 membrane binding results in reduced HOPS enrichment (Wang et al., 2003). Secondly, HOPS is required to recruit the SNARE Vam7 to sites of fusion after disassembly of post-fusion SNARE complexes; these two interactions together result in a positive feedback of recruitment of the various fusion machinery (Zick and Wickner, 2013). For intra-Golgi trafficking, knockdown of individual COG subunits in mammalian cells leads to changes in SNARE localization (Oka et al., 2004). Similarly, relocalization of the COG complex results in a redistribution of Golgi-destined vesicles, suggesting that COG recruits proper vesicles to their sites of fusion (Willett et al., 2013a). In polarized yeast exocytosis, a cluster of point mutations in the v-SNARE protein Snc2 (Snc2-M2) disrupts an interaction between the exocyst subunit Sec6 and Snc2, resulting in a mild exocyst polarization defect (Shen et al., 2013).

Another possible role for these interactions is in the protection of pre-fusion cognate SNARE complexes. HOPS competes with the disassembly machinery (Sec17/Sec18) for binding to the assembled SNARE complex and preferentially binds to trans-SNARE complexes that bridge the membranes; this protects the pre-fusion SNARE complex from premature disassembly (Collins et al., 2005; Collins and Wickner, 2007; Xu et al., 2010). Other evidence for this protective role is that HOPS prevents fusion between vacuolar compartments with non-cognate SNARE complexes, suggesting that it may “proofread” the SNARE complex prior to fusion (Starai et al., 2008). Similarly, COG binds more tightly to the assembled SNARE complex than the individual SNAREs, although a direct protective role remains to be shown (Shestakova et al., 2007). Recently, we showed that the exocyst subunit Sec6 has a tighter affinity for the Sec9:Sso1:Snc2 ternary complex than the Sec9:Sso1 binary complex (Dubuke et al., 2015); it will be interesting to see if exocyst can also proofread exocytic SNARE complexes.

In some cases, the MTCs were shown to be important for SNARE function, although the specific MTC:SNARE interactions are currently unknown. Knockdown of individual COG subunits in mammalian cells leads to an increase in uncomplexed SNAREs, and a decrease in overall SNARE protein stability (Oka et al., 2004; Fotso et al., 2005; Shestakova et al., 2007). Also in mammalian cells, SNARE assembly is reduced when GARP is depleted (Siniossoglou and Pelham, 2002). Similarly, in yeast, functional Dsl1 subunits are required for formation of the assembled t-SNARE complex, and stimulate SNARE complex assembly *in vitro* (Ren et al., 2009; Meiringer et al., 2011). These functional consequences leave open the major question of the mechanism(s) by which the various MTCs are regulating their cognate SNARE proteins.

Finally, for many of these complexes, the individual interactions were identified but not the function of these interactions in trafficking. CORVET interacts with endosomal/vacuolar SNARE proteins and SNARE complexes through the Vps33 subunit (Subramanian et al., 2004; Balderhaar et al., 2013). The Dsl1 complex interacts with t-SNARE proteins on the ER, potentially in lieu of interactions with small GTPases, and its mammalian counterpart NZR interacts with various ER-localized SNARE proteins (Kraynack et al., 2005; Meiringer et al., 2011; Tagaya et al., 2014). The GARP complex interacts with the N-terminal regulatory domain of the syntaxin homolog Tlg1, as well as the SNARE domains of several mammalian Golgi SNAREs. The mammalian GARP homolog also interacts with the assembled SNARE complex (Siniossoglou and Pelham, 2001, 2002; Conibear et al., 2003; Pérez-Victoria and Bonifacino, 2009).

Each of the “modes” of SNARE regulation by the MTCs suggests a common theme—the MTCs generally have a positive influence on cognate SNARE complex assembly. However, each mode is characterized in only a few trafficking pathways, and often only part of the information is known (e.g., binding interactions vs. functional consequences) without elucidation of the full story. Are these specific modes common across all of the trafficking pathways? Are there additional modes, waiting to be identified? How do the MTCs collaborate with the SM proteins to control the SNAREs? In many cases, experimental groundwork exists in terms of purified complexes and binding partners, indicating that quantitative *in vitro* functional assays are likely possible. Similarly, powerful genetic tools are becoming commonplace enough to begin teasing apart mechanisms in mammalian cells and other organisms that were previously characterized only in yeast. The function of the MTCs and SNAREs is an intriguing question, and by combining results from different pathways and organisms we can begin to understand the complicated interplay between these protein families in all stages of trafficking.

## Author contributions

All authors listed, have made substantial, direct, and intellectual contribution to the work, and approved it for publication.

## Funding

This work was supported by National Institutes of Health Grant GM068803 to MM.

### Conflict of interest statement

The authors declare that the research was conducted in the absence of any commercial or financial relationships that could be construed as a potential conflict of interest.

## References

[B1] Adamo J. E. Rossi G. Brennwald P. J. (1999). The Rho GTPase Rho3 has a direct role in exocytosis that is distinct from its role in actin polarity. Mol. Biol. Cell 10, 4121–4133. 10.1091/mbc.10.12.4121 10588647 PMC25747

[B2] Andag U. Neumann T. Schmitt H. D. (2001). The coatomer-interacting protein Dsl1p is required for Golgi-to-endoplasmic reticulum retrieval in yeast. J. Biol. Chem. 276, 39150–39160. 10.1074/jbc.M105833200 11493604

[B3] Archbold J. K. Whitten A. E. Hu S.-H. Collins B. M. Martin J. L. (2014). SNARE-ing the structures of Sec1/Munc18 proteins. Curr. Opin. Struct. Biol. 29, 44–51. 10.1016/j.sbi.2014.09.003 25282382

[B4] Baek K. Knödler A. Lee S. H. Zhang X. Orlando K. Zhang J.. (2010). Structure-function study of the N-terminal domain of exocyst subunit Sec3. J. Biol. Chem. 285, 10424–10433. 10.1074/jbc.M109.096966 20139078 PMC2856249

[B5] Baker R. W. Jeffrey P. D. Zick M. Phillips B. P. Wickner W. T. Hughson F. M. (2015). A direct role for the Sec1/Munc18-family protein Vps33 as a template for SNARE assembly. Science 349, 1111–1114. 10.1126/science.aac7906 26339030 PMC4727825

[B6] Balderhaar H. J. K. Lachmann J. Yavavli E. Bröcker C. Lürick A. Ungermann C. (2013). The CORVET complex promotes tethering and fusion of Rab5/Vps21-positive membranes. Proc. Natl. Acad. Sci. U.S.A. 110, 3823–3828. 10.1073/pnas.1221785110 23417307 PMC3593874

[B7] Barrowman J. Bhandari D. Reinisch K. M. Ferro-Novick S. (2010). TRAPP complexes in membrane traffic: convergence through a common Rab. Nat. Rev. Mol. Cell Biol. 11, 759–763. 10.1038/nrm2999 20966969

[B8] Bethani I. Lang T. Geumann U. Sieber J. J. Jahn R. Rizzoli S. O. (2007). The specificity of SNARE pairing in biological membranes is mediated by both proof-reading and spatial segregation. EMBO J. 26, 3981–3992. 10.1038/sj.emboj.7601820 17717530 PMC1994121

[B9] Bonifacino J. S. Hierro A. (2011). Transport according to GARP: receiving retrograde cargo at the trans-Golgi network. Trends Cell Biol. 21, 159–167. 10.1016/j.tcb.2010.11.003 21183348 PMC3073588

[B10] Brennwald P. J. Kearns B. Champion K. Keränen S. Bankaitis V. A. Novick P. J. (1994). Sec9 is a SNAP-25-like component of a yeast SNARE complex that may be the effector of Sec4 function in exocytosis. Cell 79, 245–258. 10.1016/0092-8674(94)90194-5 7954793

[B11] Bröcker C. Engelbrecht-Vandré S. Ungermann C. (2010). Multisubunit tethering complexes and their role in membrane fusion. Curr. Biol. 20, R943–R952. 10.1016/j.cub.2010.09.015 21056839

[B12] Bröcker C. Kuhlee A. Gatsogiannis C. Balderhaar H. J. K. Hönscher C. Engelbrecht-Vandré S.. (2012). Molecular architecture of the multisubunit homotypic fusion and vacuole protein sorting (HOPS) tethering complex. Proc. Natl. Acad. Sci. U.S.A. 109, 1991–1996. 10.1073/pnas.1117797109 22308417 PMC3277535

[B13] Brunet S. Sacher M. (2014). Are all multisubunit tethering complexes bona fide tethers? Traffic 15, 1282–1287. 10.1111/tra.12200 25048641

[B14] Brünger A. T. Cipriano D. J. Diao J. (2015). Towards reconstitution of membrane fusion mediated by SNAREs and other synaptic proteins. Crit. Rev. Biochem. Mol. Biol. 50, 231–241. 10.3109/10409238.2015.1023252 25788028 PMC4673598

[B15] Bryant N. J. James D. E. (2003). The Sec1p/Munc18 (SM) protein, Vps45p, cycles on and off membranes during vesicle transport. J. Cell Biol. 161, 691–696. 10.1083/jcb.200212078 12756236 PMC2199362

[B16] Cai Y. Chin H. F. Lazarova D. Menon S. Fu C. Cai H.. (2008). The structural basis for activation of the Rab Ypt1p by the TRAPP membrane-tethering complexes. Cell 133, 1202–1213. 10.1016/j.cell.2008.04.049 18585354 PMC2465810

[B17] Carr C. M. Grote E. Munson M. Hughson F. M. Novick P. J. (1999). Sec1p binds to SNARE complexes and concentrates at sites of secretion. J. Cell Biol. 146, 333–344. 10.1083/jcb.146.2.333 10427089 PMC3206579

[B18] Carr C. M. Rizo J. (2010). At the junction of SNARE and SM protein function. Curr. Opin. Cell Biol. 22, 488–495. 10.1016/j.ceb.2010.04.006 20471239 PMC2923694

[B19] Cheung P.-Y. P. Pfeffer S. R. (2016). Transport vesicle tethering at the trans golgi network: coiled coil proteins in action. Front. Cell Dev. Biol. 4:18. 10.3389/fcell.2016.00018 27014693 PMC4791371

[B20] Collins K. M. Thorngren N. L. Fratti R. A. Wickner W. T. (2005). Sec17p and HOPS, in distinct SNARE complexes, mediate SNARE complex disruption or assembly for fusion. EMBO J. 24, 1775–1786. 10.1073/pnas.1518935113 15889152 PMC1142591

[B21] Collins K. M. Wickner W. T. (2007). Trans-SNARE complex assembly and yeast vacuole membrane fusion. Proc. Natl. Acad. Sci. U.S.A. 104, 8755–8760. 10.1073/pnas.0702290104 17502611 PMC1885575

[B22] Conibear E. Cleck J. N. Stevens T. H. (2003). Vps51p mediates the association of the GARP (Vps52/53/54) complex with the late Golgi t-SNARE Tlg1p. Mol. Biol. Cell 14, 1610–1623. 10.1091/mbc.E02-10-0654 12686613 PMC153126

[B23] Conibear E. Stevens T. H. (2000). Vps52p, Vps53p, and Vps54p form a novel multisubunit complex required for protein sorting at the yeast late Golgi. Mol. Biol. Cell 11, 305–323. 10.1091/mbc.11.1.305 10637310 PMC14776

[B24] Conradt B. Shaw J. Vida T. Emr S. D. Wickner W. T. (1992). *In vitro* reactions of vacuole inheritance in *Saccharomyces cerevisiae*. J. Cell Biol. 119, 1469–1479. 10.1083/jcb.119.6.1469 1334958 PMC2289757

[B25] Costanzo M. Baryshnikova A. Bellay J. Kim Y. Spear E. D. Sevier C. S.. (2010). The genetic landscape of a cell. Science 327, 425–431. 10.1126/science.1180823 20093466 PMC5600254

[B26] Costanzo M. Baryshnikova A. Myers C. L. Andrews B. J. Boone C. (2011). Charting the genetic interaction map of a cell. Curr. Opin. Biotechnol. 22, 66–74. 10.1016/j.copbio.2010.11.001 21111604

[B27] Cottam N. P. Wilson K. M. Ng B. G. Körner C. Freeze H. H. Ungar D. (2013). Dissecting functions of the conserved oligomeric golgi tethering complex using a cell-free assay. Traffic 15, 12–21. 10.1111/tra.12128 24102787 PMC3892563

[B28] Croteau N. J. Furgason M. L. M. Devos D. Munson M. (2009). Conservation of helical bundle structure between the exocyst subunits. PLoS ONE 4:e4443. 10.1371/journal.pone.0004443 19214222 PMC2635961

[B29] Deák F. Xu Y. Chang W.-P. Dulubova I. Khvotchev M. Liu X.. (2009). Munc18-1 binding to the neuronal SNARE complex controls synaptic vesicle priming. J. Cell Biol. 184, 751–764. 10.1083/jcb.200812026 19255244 PMC2686405

[B30] Dietrich L. E. P. Boeddinghaus C. LaGrassa T. J. Ungermann C. (2003). Control of eukaryotic membrane fusion by N-terminal domains of SNARE proteins. Biochim. Biophys. Acta 1641, 111–119. 10.1016/S0167-4889(03)00094-6 12914952

[B31] Dubuke M. L. Maniatis S. Shaffer S. A. Munson M. (2015). The exocyst subunit Sec6 interacts with assembled exocytic SNARE Complexes. J. Biol. Chem. 290, 28245–28256. 10.1074/jbc.M115.673806 26446795 PMC4653681

[B32] Fasshauer D. Antonin W. Margittai M. Pabst S. Jahn R. (1999). Mixed and non-cognate SNARE complexes. Characterization of assembly and biophysical properties. J. Biol. Chem. 274, 15440–15446. 10.1074/jbc.274.22.15440 10336434

[B33] Fotso P. Koryakina Y. Pavliv O. Tsiomenko A. B. Lupashin V. V. (2005). Cog1p plays a central role in the organization of the yeast conserved oligomeric Golgi complex. J. Biol. Chem. 280, 27613–27623. 10.1074/jbc.M504597200 15932880

[B34] Fukuda M. Kanno E. Ishibashi K. Itoh T. (2008). Large scale screening for novel rab effectors reveals unexpected broad Rab binding specificity. Mol. Cell. Proteomics 7, 1031–1042. 10.1074/mcp.M700569-MCP200 18256213

[B35] Furukawa N. Mima J. (2014). Multiple and distinct strategies of yeast SNAREs to confer the specificity of membrane fusion. Sci. Rep. 4:4277. 10.1038/srep04277 24589832 PMC3940976

[B36] Garcia-Reitböck P. Anichtchik O. Bellucci A. Iovino M. Ballini C. Fineberg E.. (2010). SNARE protein redistribution and synaptic failure in a transgenic mouse model of Parkinson’s disease. Brain 133, 2032–2044. 10.1093/brain/awq132 20534649 PMC2892942

[B37] Grote E. Carr C. M. Novick P. J. (2000). Ordering the final events in yeast exocytosis. J. Cell Biol. 151, 439–452. 10.1083/jcb.151.2.439 11038189 PMC2192655

[B38] Guo W. Roth D. Walch-Solimena C. Novick P. J. (1999). The exocyst is an effector for Sec4p, targeting secretory vesicles to sites of exocytosis. EMBO J. 18, 1071–1080. 10.1093/emboj/18.4.1071 10022848 PMC1171198

[B39] Haas A. (1995). A quantitative assay to measure homotypic vacuole fusion *in vitro*. Methods Cell Sci. 17, 283–294. 10.1007/BF00986234

[B40] Hashizume K. Cheng Y.-S. Hutton J. L. Chiu C.-H. Carr C. M. (2009). Yeast Sec1p functions before and after vesicle docking. Mol. Biol. Cell 20, 4673–4685. 10.1091/mbc.E09-02-0172 19776355 PMC2777098

[B41] He B. Xi F. Zhang X. Zhang J. Guo W. (2007). Exo70 interacts with phospholipids and mediates the targeting of the exocyst to the plasma membrane. EMBO J. 26, 4053–4065. 10.1038/sj.emboj.7601834 17717527 PMC2230670

[B42] Heider M. R. Gu M. Duffy C. M. Mirza A. M. Marcotte L. L. Walls A. C.. (2016). Subunit connectivity, assembly determinants and architecture of the yeast exocyst complex. Nat. Struct. Mol. Biol. 23, 59–66. 10.1038/nsmb.3146 26656853 PMC4752824

[B43] Heider M. R. Munson M. (2012). Exorcising the exocyst complex. Traffic 13, 898–907. 10.1111/j.1600-0854.2012.01353.x 22420621 PMC3374049

[B44] Hickey C. M. Wickner W. T. (2010). HOPS initiates vacuole docking by tethering membranes before trans-SNARE complex assembly. Mol. Biol. Cell 21, 2297–2305. 10.1091/mbc.E10-01-0044 20462954 PMC2893992

[B45] Hong W. Lev S. (2014). Tethering the assembly of SNARE complexes. Trends Cell Biol. 24, 35–43. 10.1016/j.tcb.2013.09.006 24119662

[B46] Hoppins S. Collins S. R. Cassidy-Stone A. Hummel E. Devay R. M. Lackner L. L.. (2011). A mitochondrial-focused genetic interaction map reveals a scaffold-like complex required for inner membrane organization in mitochondria. J. Cell Biol. 195, 323–340. 10.1083/jcb.201107053 21987634 PMC3198156

[B47] Jahn R. Scheller R. H. (2006). SNAREs–engines for membrane fusion. Nat. Rev. Mol. Cell Biol. 7, 631–643. 10.1038/nrm2002 16912714

[B48] Jahn R. Südhof T. C. (1999). Membrane fusion and exocytosis. Annu. Rev. Biochem. 68, 863–911. 10.1146/annurev.biochem.68.1.863 10872468

[B49] Johnson R. D. Oliver P. L. Davies K. E. (2008). SNARE proteins and schizophrenia: linking synaptic and neurodevelopmental hypotheses. Acta Biochim. Pol. 55, 619–628. 18985177

[B50] Kama R. Kanneganti V. Ungermann C. Gerst J. E. (2011). The yeast Batten disease orthologue Btn1 controls endosome-Golgi retrograde transport via SNARE assembly. J. Cell Biol. 195, 203–215. 10.1083/jcb.201102115 21987636 PMC3198160

[B51] Kennedy S. Wang D. Ruvkun G. (2004). A conserved siRNA-degrading RNase negatively regulates RNA interference in C. elegans. Nature 427, 645–649. 10.1038/nature02302 14961122

[B52] Kim J. J. Lipatova Z. Segev N. (2016). TRAPP Complexes in Secretion and Autophagy. Front. Cell Dev. Biol. 4:20. 10.3389/fcell.2016.00020 27066478 PMC4811894

[B53] Klinger C. M. Klute M. J. Dacks J. B. (2013). Comparative genomic analysis of multi-subunit tethering complexes demonstrates an ancient pan-eukaryotic complement and sculpting in Apicomplexa. PLoS ONE 8:e76278. 10.1371/journal.pone.0076278 24086721 PMC3785458

[B54] Kraynack B. A. Chan A. Rosenthal E. Essid M. Umansky B. Waters M. G.. (2005). Dsl1p, Tip20p, and the novel Dsl3(Sec39) protein are required for the stability of the Q/t-SNARE complex at the endoplasmic reticulum in yeast. Mol. Biol. Cell 16, 3963–3977. 10.1091/mbc.E05-01-0056 15958492 PMC1196311

[B55] Kuhlee A. Raunser S. Ungermann C. (2015). Functional homologies in vesicle tethering. FEBS Lett. 589, 2487–2497. 10.1016/j.febslet.2015.06.001 26072291

[B56] Laufman O. Kedan A. Hong W. Lev S. (2009). Direct interaction between the COG complex and the SM protein, Sly1, is required for Golgi SNARE pairing. EMBO J. 28, 2006–2017. 10.1038/emboj.2009.168 19536132 PMC2718288

[B57] Lobingier B. T. Merz A. J. (2012). Sec1/Munc18 protein Vps33 binds to SNARE domains and the quaternary SNARE complex. Mol. Biol. Cell 23, 4611–4622. 10.1091/mbc.E12-05-0343 23051737 PMC3510022

[B58] Lobingier B. T. Nickerson D. P. Lo S.-Y. Merz A. J. (2014). SM proteins Sly1 and Vps33 co-assemble with Sec17 and SNARE complexes to oppose SNARE disassembly by Sec18. Elife 3:e02272. 10.7554/eLife.02272 24837546 PMC4060006

[B59] McNew J. A. Parlati F. Fukuda R. Johnston R. J. Paz K. Paumet F.. (2000). Compartmental specificity of cellular membrane fusion encoded in SNARE proteins. Nature 407, 153–159. 10.1038/35025000 11001046

[B60] Medine C. N. Rickman C. Chamberlain L. H. Duncan R. R. (2007). Munc18-1 prevents the formation of ectopic SNARE complexes in living cells. J. Cell Sci. 120, 4407–4415. 10.1242/jcs.020230 18057031

[B61] Medkova M. France Y. E. Coleman J. Novick P. J. (2006). The rab exchange factor Sec2p reversibly associates with the exocyst. Mol. Biol. Cell 17, 2757–2769. 10.1091/mbc.E05-10-0917 16611746 PMC1474791

[B62] Meiringer C. T. A. Rethmeier R. Auffarth K. Wilson J. Perz A. Barlowe C.. (2011). The Dsl1 protein tethering complex is a resident endoplasmic reticulum complex, which interacts with five soluble NSF (N-ethylmaleimide-sensitive factor) attachment protein receptors (SNAREs): implications for fusion and fusion regulation. J. Biol. Chem. 286, 25039–25046. 10.1074/jbc.M110.215327 21550981 PMC3137077

[B63] Miller V. J. Sharma P. Kudlyk T. A. Frost L. Rofe A. P. Watson I. J.. (2013). Molecular insights into vesicle tethering at the Golgi by the conserved oligomeric Golgi (COG) complex and the golgin TATA element modulatory factor (TMF). J. Biol. Chem. 288, 4229–4240. 10.1074/jbc.M112.426767 23239882 PMC3567674

[B64] Morgera F. Sallah M. R. Dubuke M. L. Gandhi P. Brewer D. N. Carr C. M.. (2012). Regulation of exocytosis by the exocyst subunit Sec6 and the SM protein Sec1. Mol. Biol. Cell 23, 337–346. 10.1091/mbc.E11-08-0670 22114349 PMC3258177

[B65] Munson M. Chen X. Cocina A. E. Schultz S. M. Hughson F. M. (2000). Interactions within the yeast t-SNARE Sso1p that control SNARE complex assembly. Nat. Struct. Biol. 7, 894–902. 10.1038/79659 11017200

[B66] Nicholson K. L. Munson M. Miller R. B. Filip T. J. Fairman R. Hughson F. M. (1998). Regulation of SNARE complex assembly by an N-terminal domain of the t-SNARE Sso1p. Nat. Struct. Biol. 5, 793–802. 10.1038/1834 9731774

[B67] Nordmann M. Cabrera M. Perz A. Bröcker C. Ostrowicz C. W. Engelbrecht-Vandré S.. (2010). The Mon1-Ccz1 complex is the GEF of the late endosomal Rab7 homolog Ypt7. Curr. Biol. 20, 1654–1659. 10.1016/j.cub.2010.08.002 20797862

[B68] Novick P. J. Field C. Schekman R. W. (1980). Identification of 23 complementation groups required for post-translational events in the yeast secretory pathway. Cell 21, 205–215. 10.1016/0092-8674(80)90128-2 6996832

[B69] Oka T. Ungar D. Hughson F. M. Krieger M. (2004). The COG and COPI complexes interact to control the abundance of GEARs, a subset of Golgi integral membrane proteins. Mol. Biol. Cell 15, 2423–2435. 10.1091/mbc.E03-09-0699 15004235 PMC404034

[B70] Ostrowski M. Carmo N. B. Krumeich S. Fanget I. Raposo G. Savina A.. (2010). Rab27a and Rab27b control different steps of the exosome secretion pathway. Nat. Cell Biol. 12, 19–30– sup pp 1–13. 10.1038/ncb2000 19966785

[B71] Peplowska K. Markgraf D. F. Ostrowicz C. W. Bange G. Ungermann C. (2007). The CORVET tethering complex interacts with the yeast Rab5 homolog Vps21 and is involved in endo-lysosomal biogenesis. Dev. Cell 12, 739–750. 10.1016/j.devcel.2007.03.006 17488625

[B72] Peralta E. R. Martin B. C. Edinger A. L. (2010). Differential effects of TBC1D15 and mammalian Vps39 on Rab7 activation state, lysosomal morphology, and growth factor dependence. J. Biol. Chem. 285, 16814–16821. 10.1074/jbc.M110.111633 20363736 PMC2878074

[B73] Pérez-Victoria F. J. Bonifacino J. S. (2009). Dual roles of the mammalian GARP complex in tethering and SNARE complex assembly at the trans-golgi network. Mol. Cell. Biol. 29, 5251–5263. 10.1128/MCB.00495-09 19620288 PMC2747979

[B74] Plemel R. L. Lobingier B. T. Brett C. L. Angers C. G. Nickerson D. P. Paulsel A.. (2011). Subunit organization and Rab interactions of Vps-C protein complexes that control endolysosomal membrane traffic. Mol. Biol. Cell 22, 1353–1363. 10.1091/mbc.E10-03-0260 21325627 PMC3078060

[B75] Price A. Seals D. F. Wickner W. T. Ungermann C. (2000). The docking stage of yeast vacuole fusion requires the transfer of proteins from a cis-SNARE complex to a Rab/Ypt protein. J. Cell Biol. 148, 1231–1238. 10.1083/jcb.148.6.1231 10725336 PMC2174311

[B76] Reilly B. A. Kraynack B. A. VanRheenen S. M. Waters M. G. (2001). Golgi-to-endoplasmic reticulum (ER) retrograde traffic in yeast requires Dsl1p, a component of the ER target site that interacts with a COPI coat subunit. Mol. Biol. Cell 12, 3783–3796. 10.1091/mbc.12.12.3783 11739780 PMC60755

[B77] Ren Y. Yip C. K. Tripathi A. Huie D. Jeffrey P. D. Walz T.. (2009). A structure-based mechanism for vesicle capture by the multisubunit tethering complex Dsl1. Cell 139, 1119–1129. 10.1016/j.cell.2009.11.002 20005805 PMC2806190

[B78] Sacher M. Barrowman J. Wang W. Horecka J. Zhang Y. Pypaert M.. (2001). TRAPP I implicated in the specificity of tethering in ER-to-Golgi transport. Mol. Cell 7, 433–442. 10.1016/S1097-2765(01)00190-3 11239471

[B79] Sato T. K. Rehling E. Peterson M. R. Emr S. D. (2000). Class C Vps protein complex regulates vacuolar SNARE pairing and is required for vesicle docking/fusion. Mol. Cell 6, 661–671. 10.1016/S1097-2765(00)00064-2 11030345

[B80] Schindler C. Chen Y. Pu J. Guo X. Bonifacino J. S. (2015). EARP is a multisubunit tethering complex involved in endocytic recycling. Nat. Cell Biol. 17, 639–650. 10.1038/ncb3129 25799061 PMC4417048

[B81] Schuldiner M. Collins S. R. Thompson N. J. Denic V. Bhamidipati A. Punna T.. (2005). Exploration of the function and organization of the yeast early secretory pathway through an epistatic miniarray profile. Cell 123, 507–519. 10.1016/j.cell.2005.08.031 16269340

[B82] Seals D. F. Eitzen G. Margolis N. Wickner W. T. Price A. (2000). A Ypt/Rab effector complex containing the Sec1 homolog Vps33p is required for homotypic vacuole fusion. Proc. Natl. Acad. Sci. U.S.A. 97, 9402–9407. 10.1073/pnas.97.17.9402 10944212 PMC16876

[B83] Shen D. Yuan H. Hutagalung A. H. Verma A. Kümmel D. Wu X.. (2013). The synaptobrevin homologue Snc2p recruits the exocyst to secretory vesicles by binding to Sec6p. J. Cell Biol. 202, 509–526. 10.1083/jcb.201211148 23897890 PMC3734085

[B84] Shestakova A. Suvorova E. S. Pavliv O. Khaidakova G. Lupashin V. V. (2007). Interaction of the conserved oligomeric Golgi complex with t-SNARE Syntaxin5a/Sed5 enhances intra-Golgi SNARE complex stability. J. Cell Biol. 179, 1179–1192. 10.1083/jcb.200705145 18086915 PMC2140037

[B85] Siniossoglou S. (2005). Affinity purification of Ypt6 effectors and identification of TMF/ARA160 as a Rab6 interactor. Meth. Enzymol. 403, 599–607. 10.1016/S0076-6879(05)03052-1 16473623

[B86] Siniossoglou S. Pelham H. R. B. (2001). An effector of Ypt6p binds the SNARE Tlg1p and mediates selective fusion of vesicles with late Golgi membranes. EMBO J. 20, 5991–5998. 10.1093/emboj/20.21.5991 11689439 PMC125711

[B87] Siniossoglou S. Pelham H. R. B. (2002). Vps51p links the VFT complex to the SNARE Tlg1p. J. Biol. Chem. 277, 48318–48324. 10.1074/jbc.M209428200 12377769

[B88] Sivaram M. V. S. Furgason M. L. M. Brewer D. N. Munson M. (2006). The structure of the exocyst subunit Sec6p defines a conserved architecture with diverse roles. Nat. Struct. Mol. Biol. 13, 555–556. 10.1038/nsmb1096 16699513

[B89] Spang A. (2012). The DSL1 complex: the smallest but not the least CATCHR. Traffic 13, 908–913. 10.1111/j.1600-0854.2012.01362.x 22486903

[B90] Starai V. J. Hickey C. M. Wickner W. T. (2008). HOPS proofreads the trans-SNARE complex for yeast vacuole fusion. Mol. Biol. Cell 19, 2500–2508. 10.1091/mbc.E08-01-0077 18385512 PMC2397298

[B91] Stroupe C. Collins K. M. Fratti R. A. Wickner W. T. (2006). Purification of active HOPS complex reveals its affinities for phosphoinositides and the SNARE Vam7p. EMBO J. 25, 1579–1589. 10.1038/sj.emboj.7601051 16601699 PMC1440844

[B92] Stroupe C. Hickey C. M. Mima J. Burfeind A. S. Wickner W. T. (2009). Minimal membrane docking requirements revealed by reconstitution of Rab GTPase-dependent membrane fusion from purified components. Proc. Natl. Acad. Sci. U.S.A. 106, 17626–17633. 10.1073/pnas.0903801106 19826089 PMC2764952

[B93] Struthers M. S. Shanks S. G. MacDonald C. Carpp L. N. Drozdowska A. M. Kioumourtzoglou D.. (2009). Functional homology of mammalian syntaxin 16 and yeast Tlg2p reveals a conserved regulatory mechanism. J. Cell Sci. 122, 2292–2299. 10.1242/jcs.046441 19509055 PMC2723147

[B94] Subramanian S. Woolford C. A. Jones E. W. (2004). The Sec1/Munc18 protein, Vps33p, functions at the endosome and the vacuole of *Saccharomyces cerevisiae*. Mol. Biol. Cell 15, 2593–2605. 10.1091/mbc.E03-10-0767 15047864 PMC420085

[B95] Suvorova E. S. Duden R. Lupashin V. V. (2002). The Sec34/Sec35p complex, a Ypt1p effector required for retrograde intra-Golgi trafficking, interacts with Golgi SNAREs and COPI vesicle coat proteins. J. Cell Biol. 157, 631–643. 10.1083/jcb.200111081 12011112 PMC2173848

[B96] Tagaya M. Arasaki K. Inoue H. Kimura H. (2014). Moonlighting functions of the NRZ (mammalian Dsl1) complex. Front. Cell Dev. Biol. 2:25 10.3389/fcell.2014.00025 PMC4206994 25364732

[B97] Tong A. H. Y. (2004). Global Mapping of the yeast genetic interaction network. Science 303, 808–813. 10.1126/science.1091317 14764870

[B98] VanRheenen S. M. Reilly B. A. Chamberlain S. J. Waters M. G. (2001). Dsl1p, an essential protein required for membrane traffic at the endoplasmic reticulum/golgi interface in yeast. Traffic 2, 212–231. 10.1034/j.1600-0854.2001.020307.x 11260526

[B99] Wang L. Merz A. J. Collins K. M. Wickner W. T. (2003). Hierarchy of protein assembly at the vertex ring domain for yeast vacuole docking and fusion. J. Cell Biol. 160, 365–374. 10.1083/jcb.200209095 12566429 PMC2172665

[B100] Wang W. Sacher M. Ferro-Novick S. (2000). TRAPP stimulates guanine nucleotide exchange on Ypt1p. J. Cell Biol. 151, 289–296. 10.1083/jcb.151.2.289 11038176 PMC2192651

[B101] Weber T. Zemelman B. V. McNew J. A. Westermann B. Gmachl M. Parlati F.. (1998). SNAREpins: minimal machinery for membrane fusion. Cell 92, 759–772. 10.1016/S0092-8674(00)81404-X 9529252

[B102] Weimbs T. Low S. H. Chapin S. J. Mostov K. E. Bucher P. Hofmann K. (1997). A conserved domain is present in different families of vesicular fusion proteins: a new superfamily. Proc. Natl. Acad. Sci. U.S.A. 94, 3046–3051. 10.1073/pnas.94.7.3046 9096343 PMC20319

[B103] Wiederkehr A. de Craene J.-O. Ferro-Novick S. Novick P. J. (2004). Functional specialization within a vesicle tethering complex: bypass of a subset of exocyst deletion mutants by Sec1p or Sec4p. J. Cell Biol. 167, 875–887. 10.1083/jcb.200408001 15583030 PMC2172455

[B104] Willett R. Kudlyk T. A. Pokrovskaya I. D. Schönherr R. Ungar D. Duden R. (2013a). COG complexes form spatial landmarks for distinct SNARE complexes. Nat. Commun. 4:1553 10.1038/ncomms2535 PMC3595136 23462996

[B105] Willett R. Ungar D. Lupashin V. V. (2013b). The Golgi puppet master: COG complex at center stage of membrane trafficking interactions. Histochem. Cell Biol. 140, 271–283. 10.1007/s00418-013-1117-6 23839779 PMC3748202

[B106] Wu B. Guo W. (2015). The exocyst at a glance. J. Cell Sci. 128, 2957–2964. 10.1242/jcs.156398 26240175 PMC4541039

[B107] Wu H. Turner C. Gardner J. Temple B. Brennwald P. J. (2010). The Exo70 subunit of the exocyst is an effector for both Cdc42 and Rho3 function in polarized exocytosis. Mol. Biol. Cell 21, 430–442. 10.1091/mbc.E09-06-0501 19955214 PMC2814788

[B108] Wuestehube L. J. Duden R. Eun A. Hamamoto S. Korn P. Ram R. J.. (1996). New mutants of *Saccharomyces cerevisiae* affected in the transport of proteins from the endoplasmic reticulum to the Golgi complex. Genetics 142, 393–406. 8852839 10.1093/genetics/142.2.393 PMC1206974

[B109] Wurmser A. E. Sato T. K. Emr S. D. (2000). New component of the vacuolar class C-Vps complex couples nucleotide exchange on the Ypt7 GTPase to SNARE-dependent docking and fusion. J. Cell Biol. 151, 551–562. 10.1083/jcb.151.3.551 11062257 PMC2185595

[B110] Xu H. Jun Y. Thompson J. Yates J. R. Wickner W. T. (2010). HOPS prevents the disassembly of trans-SNARE complexes by Sec17p/Sec18p during membrane fusion. EMBO J. 29, 1948–1960. 10.1038/emboj.2010.97 20473271 PMC2892374

[B111] Yamashita M. Kurokawa K. Sato Y. Yamagata A. Mimura H. Yoshikawa A.. (2010). Structural basis for the Rho- and phosphoinositide-dependent localization of the exocyst subunit Sec3. Nat. Struct. Mol. Biol. 17, 180–186. 10.1038/nsmb.1722 20062059

[B112] Yang B. Gonzalez L. C. Prekeris R. Steegmaier M. Advani R. J. Scheller R. H. (1999). SNARE interactions are not selective. Implications for membrane fusion specificity. J. Biol. Chem. 274, 5649–5653. 10.1074/jbc.274.9.5649 10026182

[B113] Yu H. Braun P. Yildirim M. A. Lemmens I. Venkatesan K. Sahalie J.. (2008). High-quality binary protein interaction map of the yeast interactome network. Science 322, 104–110. 10.1126/science.1158684 18719252 PMC2746753

[B114] Yu I.-M. Hughson F. M. (2010). Tethering factors as organizers of intracellular vesicular traffic. Annu. Rev. Cell Dev. Biol. 26, 137–156. 10.1146/annurev.cellbio.042308.113327 19575650

[B115] Zhang X. Bi E. Novick P. J. Du L. Kozminski K. G. Lipschutz J. H.. (2001). Cdc42 interacts with the exocyst and regulates polarized secretion. J. Biol. Chem. 276, 46745–46750. 10.1074/jbc.M107464200 11595741

[B116] Zhang X. Orlando K. He B. Xi F. Zhang J. Zajac A.. (2008). Membrane association and functional regulation of Sec3 by phospholipids and Cdc42. J. Cell Biol. 180, 145–158. 10.1083/jcb.200704128 18195105 PMC2213614

[B117] Zick M. Wickner W. T. (2013). The tethering complex HOPS catalyzes assembly of the soluble SNARE Vam7 into fusogenic trans-SNARE complexes. Mol. Biol. Cell 24, 3746–3753. 10.1091/mbc.E13-07-0419 24088569 PMC3843000

[B118] Zink S. Wenzel D. Wurm C. A. Schmitt H. D. (2009). A link between ER tethering and COP-I vesicle uncoating. Dev. Cell 17, 403–416. 10.1016/j.devcel.2009.07.012 19758564

[B119] Zolov S. N. Lupashin V. V. (2005). Cog3p depletion blocks vesicle-mediated Golgi retrograde trafficking in HeLa cells. J. Cell Biol. 168, 747–759. 10.1083/jcb.200412003 15728195 PMC2171815

